# DARU Journal of Pharmaceutical Sciences; The 2012 report of Editor-in-Chief

**DOI:** 10.1186/1560-8115-20-1

**Published:** 2012-07-19

**Authors:** Mohammad Abdollahi

**Affiliations:** 1 Faculty of Pharmacy, and Pharmaceutical Sciences Research Center, Tehran University of Medical Sciences, Tehran, Iran

## 

DARU Journal of Pharmaceutical Sciences (DARU) is one of the 47 scientific journals of Tehran University of Medical Sciences (TUMS). When the journal was launched in 1991, articles were published in the Persian language; English abstracts were then added in 1995, and finally it completed the transition to publishing in the English language for both abstracts and full-text in early 1999.

The main scope of this journal is to serve as a platform for all areas of drug conception, design, manufacturing, classification and assessment. DARU welcomes outstanding studies from basic research to clinical investigations as original papers, systematic reviews, meta-analyses, general reviews, mini-reviews, short communications, and editorials from the global scientific community.

DARU considers guidelines provided by International Committee of Medical Journal Editors (http://www.icmje.org), Committee on Publication Ethics (COPE; http://www.publicationethics.org), World Association of Medical Editors (WAME; http://wame.org), and European Association of Science Editors (EASE; http://www.ease.org.uk) and request authors to meet these guidelines in submitting their manuscripts. For clinical trials, this journal requires registration in a public trials registry also.

**Figure 1:**
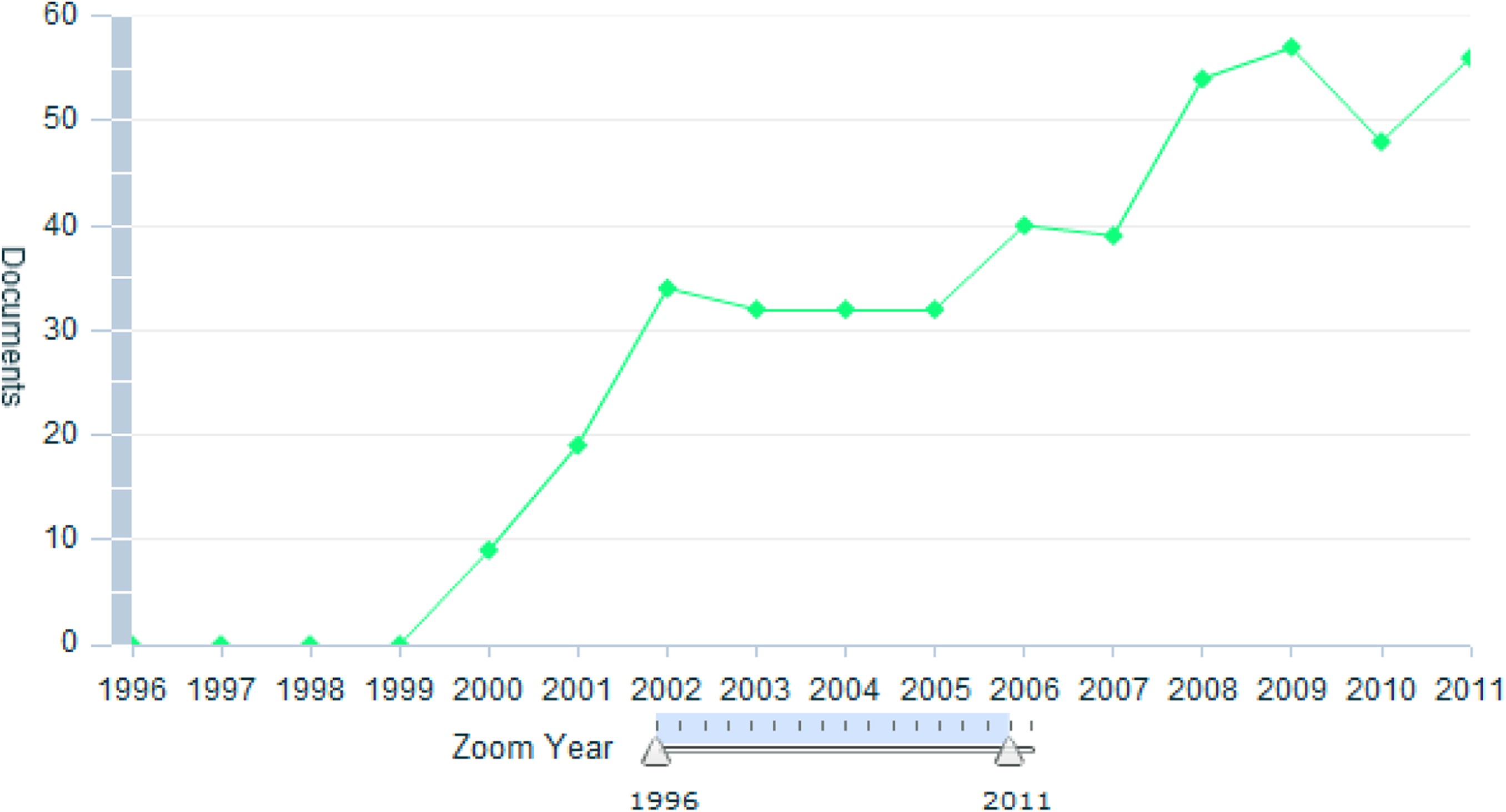
**Growth of DARU published papers in Scopus**®.

**Figure 2:**
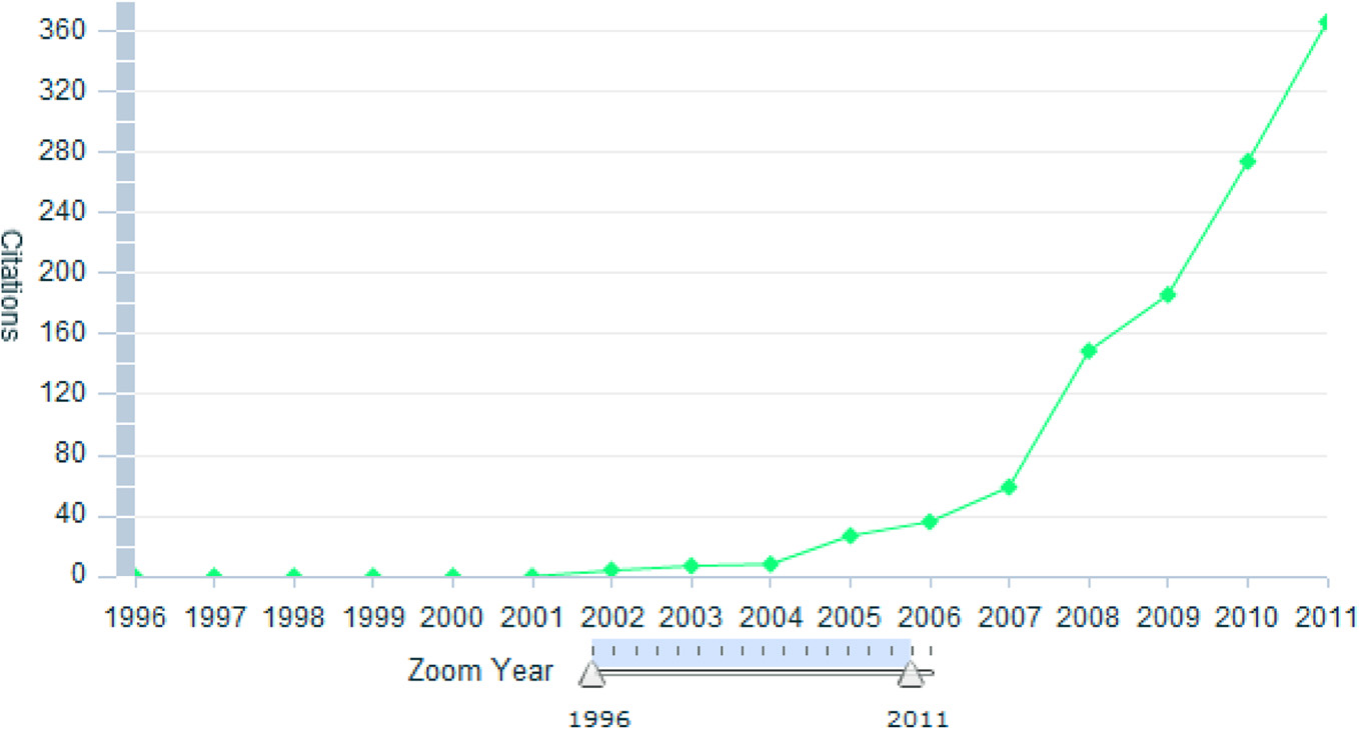
**Number of citations to DARU published papers in Scopus®**.

The Editor-in-Chief and the journal DARU are members of these three ethical organizations. The journal is a favorite of many researchers in the field of pharmacy and has been free to all readers in the world since its launch. Initiatives to extend the reach of the journal to readers outside the academic community, framed by DARU policy to promote the discipline of drug fields have been successful, judged by rate of highly cited papers and contribution of scientists in different fields from various departments. Scopus® data (Figures 1 and 2, Table 1 ) indicate that this journal has had both immediate impact and an enduring quality. The 2011 impact factor and h-index of DARU according to ISI Web of Science is 0.625 and 8, respectively.

**Table 1:** List of top 10 most cited articles according to Scopus

**Reference**	**No. of Citation**
Krishna Mohan et al. 2007 [1]	25
Sajjadi 2006 [2]	25
Delazar et al. 2006 [3]	23
Patel et al. 2006 [4]	21
Abdollahi et al. 2008 [5]	17
Miri et al. 2008 [6]	17
Delazar et al. 2004 [7]	16
Khorram Khorshid et al. 2008 [8]	15
Masoompour et al. 2008 [9]	14
Yaghoubi et al. 2007 [10]	14

In early 2012, DARU moved to Springer BioMed Central for publication as an Open Access journal. In the new Open Access model, all articles are freely and permanently accessible online immediately upon publication, without subscription charges or its barriers. Authors of articles published in DARU are the copyright holders of their articles. Following publication in DARU the full-text of each article is deposited immediately and permanently in repositories in e-Depot, the National Library of the Netherlands' digital archive of electronic publications. In Open Access journals, authors are asked to pay article processing charges, of course in DARU, TUMS as the owner of the journal will pay on behalf of authors for the first 3 years of its publishing with Springer BioMed Central and thus all authors are waived in this period of time.

The editorial policy of this journal is that all submitted manuscripts are initially examined by the Editor-in-Chief and if passed initial requirements, then one of the Section Editors will be assigned to handle its peer review process. Main initial requirements include novelty of the study and paper, fitting in scope of journal, and full adherence to instruction guideline and ethical principles.

Statistics for the year 2012 indicates that until May 24, 2012, a total of 272 submissions were received, of which, 73 entered to peer review process, 32 (44%) were accepted and 41 (56%) were rejected. The average review time has been 23 days.

DARU is indexed and abstracted in many abstracting and indexing databases such as ISI Web of Science, PubMed Central, Scopus, Excerpta Medica/EMBASE, Scirus, Directory of Open Access Journals (DOAJ), Biological Abstracts (BIOSIS), EBSCO, Asian Science Citation Network (ASCN), CAB Abstracts, Chemical Abstracts Service (CAS), Free Medical Journals, Global Health, Google Scholar, HINARI, Index Copernicus, Index Medicus for WHO Eastern Mediterranean Region (IMEMR), International Pharmaceutical Abstracts (IPA), Natural Product Extracts (NAPRALERT), Open J-Gate, SIR.

Publication as the final important stage of any scientific research is necessary for development of sciences and knowledge of the world. By publication, world scientists talk to each other and share their idea, thoughts, findings, and opinions. The science of pharmacy is not an exclusive and thus we need all scientists of the world to consider this journal as one of their favorite to share their studies with others. The editorial board and managers of this journal try their best to develop knowledge and technology in the field of pharmacy in the globe by adhering higher standards of editing and publication to meet the needs of academics, researchers and professionals.
